# The Association of Social Capital and Self-Rated Health Between Urban Residents and Urbanized Rural Residents in Southwest China

**DOI:** 10.3389/fpubh.2021.718793

**Published:** 2021-08-25

**Authors:** Tianpei Ma, Bo Gao

**Affiliations:** ^1^Laboratory for Aging and Cancer Research, National Clinical Research Center for Geriatrics, West China Hospital, Sichuan University, Chengdu, China; ^2^Department of Health Related Social and Behavioral Science, West China School of Public Health and West China Fourth Hospital, Sichuan University, Chengdu, China

**Keywords:** social capital, self-rated health, urbanization, urbanized rural residents, Southwest China

## Abstract

China has seen an accelerated process of urbanization in the past 30 years. The influence of urbanization on health is complex and primarily influenced by changes in social capital. The purpose of this research was to compare the social capital between urban residents and urbanized rural residents of southwest China and its relationship with self-rated health. It is of great significance to study the difference of social capital between urban and urbanized rural residents to help urbanized rural residents improve their social adaptability and health. Data was collected from 1,646 residents between November and December of 2017 in Chengdu. Three logistic regressions were used to investigate the association between social capital and self-rated health by controlling for demographic variables, lifestyles factors, and health status factors. We observed that urban residents' self-rated health had a higher proportion of “good” than that of urbanized rural residents (*P* = 0.017). After controlling for factors such as health status and demographic characteristics, participants with higher social capital had better self-rated health. Urbanized rural residents with higher community trust and belonging had better self-rated health (OR = 0.701, 95% CI = 0.503~0.978), however urban residents with higher personal social networks and family relationships had better self-rated health (OR = 0.676, 95% CI = 0.490~0.933 and OR = 0.666, 95% CI = 0.450~0.987, respectively). Different types of communities should focus on the types of social capital from different sources, so as to take more targeted measures to improve the social support of residents and improve their health. Improving residents' social trust and sense of belonging may help urbanized rural residents better adapt to the new living environment and help them complete the identity transformation.

## Introduction

In the past 30 years, China has seen an accelerated process of urbanization (1). In general, urbanization is a form of migration of a country's population from rural to urban areas (2). The expansion of Chinese cities has been dramatic. In 2011, the proportion of the urban population (51.3%) exceeded that of the rural population for the first time in history (3). In 2020, China's urbanization rate has reached 63.9%. Due to the rapidity of urbanization, in many rural areas that have become urban districts. The impact of urbanization on individual health outcomes has both positive and negative consequences. On the one hand, changes in living environment and lifestyle are all related to the rapid growth of urbanization (4). Many factors, such as deteriorating air quality, increased high-calorie intake, and reduced social interaction with neighbors, affect the health of the residents. On the other hand, people tend to have better access to quality health services and other community resources, including health information (5). The influence of urbanization on health is complex and primarily influenced by changes in reciprocity and trust, social structure and networks, which are mentioned in most definitions of social capital (6).

Social capital has been defined as “those features of social organization that facilitate cooperation for mutual benefit, such as the extent of interpersonal trust between citizens, norms of reciprocity, and density of civic associations” (7, 8). More and more researches have been conducted on the role of social capital on personal health in these two decades (9). A study based on data from 39 states in US found that lack of social capital was strongly correlated with higher total mortality, death rate of heart disease and infant mortality (10). Moreover, a review illustrates that the association between social capital and personal health outcome may differ depending on the specific aspects of social capital being explored (11).

There are many indicators for evaluating the health of the population, including physical health, mental health and disease status. Various assessments of health status frequently ask respondents to rate their overall health with the categories of excellent, good, fair, and poor (12). Self-rated health appears to have a significant, independent association with mortality risks in numerous studies, when included medical, behavioral, or other health-related indicators (13). Ou et al. found that poor self-rated health was related to premature mortality and chronic health conditions (14). In addition, self-rated health is susceptible to many external factors, such as material, psychosocial, behavioral, workplace environmental and social capital (15, 16). A substantial body of literature assessed on the association of social capital on individuals' self-rated health in developed countries. For instance, Kawachi and colleagues detected a contextual effect of low social capital on the risk of poor self-rated health among US residents, after controlling for certain individual-level factors, such as low income, low education, smoking (7). Snelgrove showed that a protective relationship with current self-rated health and social trust after adjusting for individual characteristics, baseline self-rated health and individual social trust in Britain (17). Nevertheless, a few researches paid attention to the influence of social capital on self-rated health among Chinese residents. Using multilevel analysis, Meng et al. found that trust in social capital indicators was beneficial to self-rated health in China (6). Research by Zhu et al. showed that there is inequality in objective health outcomes between the floating population and local hypertensive patients, but there is no inequality in subjective health outcomes (18). These studies have examined the relationship between social capital and self-rated health in urban or rural areas. Taking into account the particularity of China's urban-rural dual structure, comparisons between different groups of people may help us better understand the impact of social capital on China's self-rated health.

In the context of China's urbanization policy, the government collectively collects the farm land that was originally cultivated by rural residents in the villages around the city. These residents were concentrated in the apartment community and their household registration status was converted from rural to urban (19). These are called urbanized rural residents (3). They left the original land and faced the problem of re-establishing a living circle. The lives of these policy immigrants have changed in many ways compared to their original lives. There are three main changes: first, their careers were no longer farmers. But because of their low level of education and lack of necessary work skills, this created a serious economic burden. Second, the type of social medical insurance had changed. There were some differences in the payment standard, reimbursement ratio and reimbursement scope between rural residents' medical insurance and urban residents' medical insurance. Third, their living space changed from scattered brick house to single-family high-rise apartments. Urbanized rural residents may experience challenge due to changes in their living environment and lifestyle. As rural-to-urban migration may influence migrants' health or well-being by exposing to new environmental risk and benefits, stimulating changes in patterns of behavior and connection of social network, and providing access to resources which were unavailable at the original place (20, 21). In the new living environment, how to help them establish new social connections and social support and improve their health level has become an urgent problem to be solved.

However, in the background of China's urbanization development, empirical evidence on the relationship between social capital and self-rated health is very limited (22). This study mainly addresses two objectives: first, to study the health status of urbanized rural residents and the differences among urbanized rural residents and urban residents. Second, to explore the differences in the relationship between social capital and self-rated health of these two groups?

## Materials and Methods

### Study Setting

Our cross-sectional study was conducted between November and December of 2017. The face-to-face questionnaire survey was conducted in the community of Chengdu, which is one of the most developed cities in Southwest China. As the urbanization speed of Chengdu city continues to accelerate, a large number of urbanized rural residents swarm into the city, which poses many challenges to urban management. Meanwhile, it also provides a good opportunity and conditions for us to study its social capital and health status. Since 2004, Chengdu has transferred an average of nearly 200,000 farmers to cities and towns each year, and the city has built a total of 630 farmers' concentrated residential areas and new rural communities, with a total area of more than 28 million square meters (23). Urbanized rural residents are important human resource in the process of urbanization. Establishing and improving public services such as education, culture, and medical care, and the old-age security and employment security systems are of great significance to help urbanized rural residents better integrate into urban life and promote social equity. Chengdu's reform experience has important implications for urbanization development policies in other regions of the country.

### Study Participants

The selection criteria for the respondents were: (1) 15 years of age and older; (2) residents who lived in the selected community for half a year or more (to exclude some temporarily rented residents); (3) no mental illness and hearing impairment, able to express themselves; (4) respondents must answer the questions themselves; (5) urbanized rural resident: their land were levied because of the urbanization policy and their rural hukou (official registration) were converted into urban hukou within 15 years. Urban residents: their urban hukou period were more than 15 years because of urban planning.

### Sampling and Sample Size

The survey used a multi-stage stratified random sampling method. First, Chengdu was divided into central urban areas and suburbs according to economic level, and one district was randomly selected in the central urban area and the suburbs. Then, we randomly selected an urban residential community and an urbanized rural residential resettlement community in each district, and uniformly codes the buildings in the selected community. According to the family, we surveyed all eligible family members present in each household.

We calculated the sample size using the following formula: *n* = [μ^2^α/2π (1-π)]/δ^2^ (24), where π = 33.2% [which was 2-week prevalence rate in the population aged 15 and over in the fifth National Health Service Survey of Sichuan Province in 2013 (25)], δ = 1.5% (δ is the allowable error, determined by the researcher based on previous experience), α = 0.05, μ^2^α/2 = 1.96. Based on this formula, the sample size was calculated to be 1,739. A total of 1,740 community residents were surveyed face-to-face by trained investigators. Excluding questionnaires with too much missing information, 1,646 valid questionnaires were finally obtained, and the effective rate was 94.6%. The study protocol was approved by the Institutional Review Board of School of Public Health, Sichuan University. Informed consent was obtained from each participant following a detail explanation about the purpose of the study.

### Variables

The questionnaire included three parts, (a) demographic characteristics, health-related factors, and health status, (b) social capital, (c) self-rated health.

#### Demographic Characteristics, Health-Related Factors, and Health Status

Demographic characteristics mainly included gender, age, marital status, education, income, and employment status. Health-related factors was considered to include these questions, “Are you currently smoking?,” “Are you currently drinking alcohol?,” and “How many times have you exercised on average every week for the past 6 months?.” Health status of participants was measured by two indices, the past 2 weeks of any diseases, and diagnosis of chronic diseases (0 = No, 1 = Yes). History of chronic diseases included hypertension, diabetes, chronic bronchitis, chronic gastritis, coronary heart disease, rheumatism and other diseases.

#### Social Capital Measurement

There is an ongoing debate about how to measure social capital. On the basis of a large number of theoretical studies on social capital in the early stage, we referred to domestic and foreign measure instruments (26, 27), and formed this social capital scale through the Delphi method. The questionnaire of social capital (see Supplementary Table 1) has 23 items, divided into five domains, Personal Social Network (SC1, 4 items), Interpersonal Support (SC2, 4 items), Family Relationship (SC3, 5 items), Community Participation (SC4, 3 items), Community Trust and Belonging (SC5, 7 items). SC1 mainly measures the number of people who are close to each other in daily life and economics, and the number of social activities with them. SC2 represents the support of others, for example, “when you are sick or uncomfortable, can you always get the care of others?” SC3 indicates whether family relationships are harmonious, including relationships with spouses, parents, and children. SC4 measures the individual's community participation, such as “The number of times you have participated in a community activity in the last year.” SC5 represents the individual's sense of belonging to the community and the trust of the community residents. For example, “If you have to move away from where you live now, do you feel uncomfortable?” (Specific items for the Social Capital Scale and scoring methods are provided in Supplementary Table 1.) The answers consisted of 2-, 4-, and 5-point Likert scales, with the higher score indicating a higher level of social capital. The sum of the scores for all items in each domain was the score for that domain. Each respondent's SC1, SC2, SC3, SC4, and SC5 scores were dichotomized by the cutoff point of the median of the corresponding social capital scores: scores lower than median scores meant low social capital. The reliability of this scale can be acceptable (Cronbach's alpha 0.681, SC1 = 0.539, SC2 = 0.602, SC3 = 0.609, SC4 = 0.411 and SC5 = 0.788).

#### Self-Rated Health

We used an item to reflect the self-rated health of the respondents: would you say that in general your health is excellent, very good, good, fair, or poor? From this question, we created a dichotomous outcome measure (0 = excellent, very good, or good; 1 = fair or poor) (7).

### Data Quality Control

During the data collection phase, undergraduate or graduate students with a medical background were selected as investigators, and they were trained intensively before the survey. After the daily survey, the investigator cross-checked the questionnaire on that day and signed and confirmed it. At the data collation and analysis stage, the verifier cleaned up the database and deleted missing records.

### Statistical Analysis

The database was set up with EpiData 3.0 (Denmark). Descriptive statistics were used to illustrate demographic characteristics of participants. And we used chi-square test to undertake an analysis of participants' social capital by each indicator of demographic characteristics. Logistic regression was used to describe the relationship between social capital and self-rated health by controlling for demographic variables. In the first model (Model 1), odds ratios (ORs) and 95% confidence intervals (95% CIs) were calculated for the relationship of five dimensions of social capital and self-rated health. Model 1 only included five dimensions of social capital as independent variables. In the second model (Model 2), the OR (95% CI) was adjusted by controlling for demographic variables, including gender, age, education, marital status, income, employment status. The third model (Model 3) controlled the health-related factors and health status based on Model 2, including smoking, drinking, physical exercise, the past 2 weeks of any diseases, and chronic diseases. We used the forward method to filter variables. The social capital contents were also considered in Model 2 and Model 3. We used the forward method to filter variables. **Tables 3**, **4** showed the variables that eventually enter the model. The goodness of fit about these models were estimated by Hosmer-Lemeshow test (see Supplementary Table 5). All statistical analyses were performed with IBM SPSS 21.0. P < 0.05 was considered to indicate a statistically significant difference.

## Results

### Characteristics of the Participants

The descriptive information of 1,646 participants were presented in Table 1. There were 885 urban residents (53.8%) and 761 urbanized rural residents (46.2%). The average age of urban residents and urbanized rural residents was 55.6 years (SD = 16.9) and 58.7 years (SD = 14.9). More than half of the respondents were women. The vast majority of the participants were married (80.6 and 76.3%, respectively). Urbanized rural residents reported lower education levels and income than urban residents. More than half of the urbanized rural residents were unemployed (62.7%).

**Table 1:** Characteristics of the participants.

	**Total (%)**	**Urban residents (%)**	**Urbanized rural residents (%)**	**χ^2^**	***P***
Gender				18.498	<0.001^**^
Male	511(31.0)	315(35.6)	196(25.8)		
Female	1,135(69.0)	570(64.4)	565(74.2)		
Age(years)				23.089	<0.001^**^
<45	214(13.0)	147(16.6)	67(8.8)		
45–54	422(25.6)	215(24.3)	207(27.2)		
55–64	389(23.6)	209(23.6)	180(23.7)		
65+	621(37.7)	314(35.5)	307(40.3)		
Marital status				35.017	<0.001^**^
Single	100(6.1)	69(7.8)	31(4.1)		
Married	1,294(78.6)	713(80.6)	581(76.3)		
Divorced	35(2.1)	22(2.5)	13(1.7)		
Widowed	217(13.2)	81(9.2)	136(17.9)		
Education				240.193	<0.001^**^
Primary school and below	635(38.6)	218(24.6)	417(54.8)		
Secondary school	473(28.7)	243(27.5)	230(30.2)		
High school	297(18.0)	219(24.7)	78(10.2)		
College and above	241(14.6)	205(23.2)	36(4.7)		
Personal monthly income (CNY)				297.404	<0.001^**^
<2,000	965(58.6)	348(39.3)	617(81.1)		
2,000~	230(14.0)	174(19.7)	56(7.4)		
3,000~	184(11.2)	141(15.9)	43(5.7)		
4,000+	267(16.2)	222(25.1)	45(5.9)		
Employment status				223.205	<0.001^**^
Employed	470(28.6)	309(34.9)	161(21.2)		
Retired	464(28.2)	341(38.5)	123(16.2)		
Unemployed	712(43.3)	235(26.6)	477(62.7)		

** *P < 0.001*.

### The Distribution of Health-Related Factors, Self-Rated Health and Social Capital

The current smoking and drinking behavior were not significantly different between the two groups of participants (*P* > 0.05), while it seemed better for urbanized rural residents to participate in physical exercise every week (*P* = 0.036). It can be seen from the chronic disease and 2-week illness that the health status of urban residents was better than that of urbanized rural residents (Table 2). The distribution of self-rated health was different between urban residents and urbanized rural residents (*P* = 0.017). The self-rated health of urbanized rural residents was worse than that of urban residents. By comparing the five dimensions of social capital, it showed that the urban residents had better family relationships (*P* = 0.019). However, the community trust and sense of belonging of urbanized rural residents was higher (*P* < 0.001).

**Table 2:** The distribution of health-related factors, self-rated health and social capital between urban residents and urbanized rural residents.

	**Urban residents (%)**	**Urbanized rural residents (%)**	**χ^2^**	***P***
Current smoker			0.262	0.626
No	698(78.9)	608(79.9)		
Yes	187(21.1)	153(20.1)		
Current drinker			1.028	0.322
No	701(79.2)	618(81.2)		
Yes	184(20.8)	143(18.8)		
Exercise/week(times)			6.635	0.036^*^
6+	550(62.1)	518(68.1)		
1~5	153(17.3)	105(13.8)		
<1	182(20.6)	138(18.1)		
Chronic disease			4.147	0.045^*^
No	587(66.3)	468(61.5)		
Yes	298(33.7)	293(38.5)		
Ill within the past 2 weeks			3.945	0.047^*^
No	604(68.2)	484(63.6)		
Yes	281(31.8)	277(36.4)		
Self-rated health			5.823	0.017^*^
Good	626(70.7)	496(65.2)		
Bad	259(29.3)	265(34.8)		
SC1			0.923	0.348
High	443(50.1)	399(52.4)		
Low	442(49.9)	362(47.6)		
SC2			1.627	0.213
High	484(54.7)	440(57.8)		
Low	401(45.3)	321(42.2)		
SC3			5.589	0.019^*^
High	520(58.8)	403(53.0)		
Low	365(41.2)	358(47.0)		
SC4			2.454	0.125
High	475(53.7)	379(49.8)		
Low	410(46.3)	382(50.2)		
SC5			15.131	<0.001^**^
High	408(46.1)	424(55.7)		
Low	477(53.9)	337(44.3)		

* *P < 0.05*,

** *P < 0.001*.

*SC1: Personal Social Network, SC2: Interpersonal Support, SC3: Family Relationship, SC4: Community Participation, SC5: Community Trust and Belonging. Use the median as a criterion for dividing high and low group of social capital*.

### Associations Between Social Capital and Self-Rated Health

The logistic regression models were established with self-rated health as the dependent variable, social capital, demographic characteristics and health status factors as independent variables. After testing, the VIF values between social capital and other socio-economic factors were <10, and there was no collinearity between the variables. The relationships between social capital and self-rated health in different logistic regression models are presented in Tables 3, 4 among urbanized rural residents and urban residents. For urbanized rural residents, higher SC5 was significantly associated with self-rated health in Model 2 and Model 3 (OR = 0.676, 95% CI = 0.491~0.931 and OR = 0.701, 95% CI = 0.503~0.978, respectively). For urban residents, SC1 was significantly associated with self-rated health all three models. People with higher SC1 had better self-rated health in Mode 1 (OR = 0.683, 95% CI = 0.505~0.923), Model 2 (OR = 0.669, 95% CI 0.487~0.918), and Model 3 (OR = 0.676, 95% CI = 0.490~0.933). In addition, SC3 was also protective factors after controlling variables of demographic characteristics and health status (OR = 0.598, 95% CI = 0.407~0.878 in Model 2 and OR = 0.666, 95% CI = 0.450~0.987 in Model 3).

**Table 3:** Associations of social capital to self-rated health among urbanized rural residents.

	**Model 1**			**Model 2**			**Model 3**		
	**OR**	**95% CI**	***P***	**OR**	**95% CI**	***P***	**OR**	**95% CI**	***P***
**SC1(Low)**									
High	1.083	0.792, 1.481	0.617	1.056	0.770,1.448	0.736	1.085	0.781,1.507	0.626
**SC2(Low)**									
High	0.821	0.598,1.128	0.223	0.774	0.560, 1.068	0.119	0.778	0.557, 1.088	0.142
**SC3(Low)**									
High	0.775	0.568, 1.056	0.107	0.872	0.627, 1.212	0.413	0.951	0.674, 1.341	0.775
**SC4(Low)**									
High	1.115	0.821, 1.515	0.487	1.113	0.816, 1.519	0.497	1.119	0.810, 1.547	0.495
**SC5(Low)**									
High	0.740	0.542, 1.012	0.060	0.676	0.491, 0.931	0.017^*^	0.701	0.503, 0.978	0.036^*^
**Age (<45)**									
45-54				1.691	0.872, 3.280	0.120	1.352	0.688, 2.657	0.381
55~64				2.702	1.393, 5.239	0.003^*^	1.639	0.824, 3.261	0.159
65+				2.453	1.297, 4.641	0.006^*^	1.420	0.729, 2.767	0.303
**Ill within the past 2 weeks (No)**									
Yes							1.904	1.328, 2.731	<0.001^*^
**Chronic disease (No)**									
Yes							2.204	1.521, 3.194	<0.001^*^

*SC1: Personal Social Network, SC2: Interpersonal Support, SC3: Family Relationship, SC4: Community Participation, SC5: Community Trust and Belonging. Model 1 only included five dimensions of social capital as independent variables. Model 2 controlled the influence of demographic characteristics (gender, age, marital status, education, income, and employment status), and Model 3 adjusted for risk factors (2-week illness, chronic diseases, smoking, drinking, and physical exercise) based on Model 2. Use the median as a criterion for dividing high and low group of social capital*.

* *P < 0.05. The control group is marked in brackets*.

**Table 4:** Associations of social capital to self-rated health among urban residents.

	**Model 1**			**Model 2**			**Model 3**		
	**OR**	**95% CI**	***P***	**OR**	**95% CI**	***P***	**OR**	**95% CI**	***P***
**SC1(Low)**									
High	0.683	0.505,0.923	0.013^*^	0.669	0.487,0.918	0.013^*^	0.676	0.490,0.933	0.017^*^
**SC2(Low)**
High	0.745	0.548,1.013	0.060	0.812	0.588, 1.121	0.205	0.804	0.579, 1.116	0.192
**SC3(low)**
High	0.757	0.559, 1.027	0.073	0.598	0.407, 0.878	0.009^*^	0.666	0.450, 0.987	0.043^*^
**SC4(low)**
High	1.174	0.866, 1.593	0.302	1.105	0.800, 1.526	0.545	1.081	0.778, 1.501	0.642
**SC5(low)**
High	1.006	0.747, 1.354	0.971	0.832	0.604, 1.146	0.260	0.830	0.599, 1.150	0.262
**Gender (male)**
Female				1.479	1.039, 2.106	0.030^*^	1.458	1.019, 2.086	0.039^*^
Age (<45)									
45–54				0.516	0.301, 0.883	0.016^*^	0.473	0.274, 0.818	0.007^*^
55–64				0.449	0.237, 0.853	0.014^*^	0.347	0.179, 0.671	0.002^*^
65+				0.765	0.410, 1.427	0.400	0.532	0.278, 1.018	0.057
**Marital status (no)**
Yes				1.991	1.231, 3.220	0.005^*^	1.981	1.216, 3.229	0.006^*^
**Employment status (employed)**
Retired				2.509	1.446, 4.354	0.001^*^	2.257	1.293, 3.940	0.004^*^
Unemployed				2.015	1.040, 3.902	0.038^*^	1.909	0.980, 3.719	0.057
**Income (<2,000)**
2,000~				2.110	1.209, 3.685	0.009^*^	1.995	1.133, 3.512	0.017^*^
3,000~				1.166	0.645, 2.110	0.611	1.121	0.614, 2.046	0.711
4,000+				1.400	0.783, 2.503	0.257	1.302	0.724, 2.339	0.378
**Ill within the past 2 weeks (No)**
Yes							1.465	1.030, 2.084	0.034^*^
**Chronic disease (No)**
Yes							1.951	1.337, 2.847	0.001^*^

*SC1: Personal Social Network, SC2: Interpersonal Support, SC3: Family Relationship, SC4: Community Participation, SC5: Community Trust and Belonging. Model 1 only included five dimensions of social capital as independent variables. Model 2 controlled the influence of demographic characteristics (gender, age, marital status, education, income, and employment status), and Model 3 adjusted for risk factors (2-week illness, chronic diseases, smoking, drinking, and physical exercise) based on Model 2. Use the median as a criterion for dividing high and low group of social capital*.

* *P < 0.05. The control group is marked in brackets*.

## Discussion

This research is dedicated to exploring social capital among urban residents and urbanized rural residents of West China and its relationship with self-rated health.

In the past 10 years, China's urbanization process has been very rapid, and Chengdu is also undergoing a process of rapid urbanization. Chengdu, the capital city of Sichuan Province, has a population of 17 million permanent residents, of which nearly 2 million are migrants. At present, the urbanization rate in Chengdu is 71.9% (28). Urbanized rural residents do not have urban hukou or have obtained urban hukou in recent years. The urban health service facilities and subsidized health care were better than the rural areas under the urban-rural dual structure (29). We found that urban residents and urbanized rural residents have statistically significant differences in 2-week illness, chronic disease, and self-rated health. The health status of urban residents was better than that of urbanized rural residents. And urbanized rural residents' self-rated health had a lower proportion of “good.” Many literatures have reported that socioeconomic status (30), health status (31), health-related behaviors (32), social capital (33) and other factors may have an impact on self-rated health. The differences between these two types of residents may cause differences in their own health assessments.

By investigating the social capital of residents, we found that the community trust and belonging of urbanized rural residents were better than those of urban residents. It showed that these participants had maintained the original community contact, and the resettlement community was mostly an acquaintance. However, the family relationship of urbanized rural residents was not so close, which was reflected in the relationship with parents and the relationship between husband and wife was lower than that of urban residents. In the urbanization process, the residential mode of centralized resettlement changed the former courtyard-style decentralized living mode, which led to the decomposition of the original joint family into a nuclear family, which may also weaken the intergenerational relationship. In addition, urbanized rural residents faced new life issues arising from urbanization, including employment, medical care, education for their children, etc. Due to the pressure of life, their time spent with their spouses and parents had decreased, leading to family tensions (34). This suggests that policies should pay more attention to various social insurance issues of urbanized rural residents to alleviate their living pressure and promote the stability of their family relationships.

Our study showed that a significant positive association between social capital and self-rated health. After controlling for factors such as health risk factors and demographic characteristics, participants with higher social capital had better self-rated health. The social capital factor that affects the self-rated health of urbanized rural residents was mainly the sense of community trust and belonging. In the former rural life in China, the neighborhood relationship between the residents was very close, and the neighbors often exchanged or helped each other. In urbanized communities, most residents in the same settlement were former neighbors, so their community belonging and trust were still significantly related to self-reported health. However, modern urban life may reconstruct their social networks after they moved in. These urbanized rural residents may tend to decrease social interaction and feel lonely or isolated as they moved into modern or high-rise apartment (35). It should be noted that the proportion of urbanized rural residents who are over 65 years old reaches 40.3%. Most of them do not have formal and stable work. Their focus of life is mainly in the communities where they live, so they have a strong dependence on the community environment. As a large marginal population concluding on unemployed rural migrants has been created, the urban community area will become an important resource of social capital for them in providing neighborhood-based mutual help or job information (36). The lack of neighboring relationships and long-term isolation, loneliness, the pressure of life may affect their assessment of health (37, 38). Schultz et al. found that social capital measures, such as informal socializing, formal group involvement, organized group interaction and volunteer activity, were the significant predictor of self-rated health (39). Evidence from an urban renewal scheme in Hong Kong has shown that the establishment of good community policing and affinity neighborhood committees can greatly enhance residents' trust and well-being in the community (38). By carrying out various meaningful community activities, enhancing the emotional exchanges between residents, increasing the community participation of residents, and creating a family atmosphere for them, they can feel more social support and social trust, reduce the pressure on life, and thus enhance individual health (17).

However, we found that the social capital factors that affect the self-rated health of urban residents were primarily personal social networks and family relationships, which referred to individual-level social capital. Different from urbanized rural communities, residents' neighborhood relationships were relatively stable in the urban community. Most urban residents were forced to put most of their energy into their work, and rarely had time to communicate with their neighbors. Their sources of social support and social networks were more extensive from family, friends, and associates, but less on social capital at the community level (40). The findings from the current multivariate analysis showed that the relationship between individual social capital and health outcome had backing from other studies (41). For example, an analysis from older Americans found that social networks were associated with a lower presence of depressive symptoms (42). Verhaeghe et al. suggested there was a positive relationship between network social capital and self-rated health, and social connections from different classes provided people different sets of resources. Network social capital from strong ties was more important to self-rated health than network social capital from weak ties (43). Generally, urban residents had higher socioeconomic status, and social capital at individual level such as personal social networks and family relationships, was stronger social capital for them. Their self-rated health was more strongly affected by individual social capital. In addition, we found that, after controlling for other factors, marital status, employment status, and income of urban residents were significantly associated with self-rated health, while this relationship was not significant for urbanized rural residents. This also implied that socioeconomic factors had an important impact on self-rated health of urban residents.

The main limitation of this study is that it is a cross-sectional survey, it does not validate the causal relationship between social capital and self-rated health. Therefore, prospective researches are needed to confirm our finding. In addition, the questionnaire for measuring social capital was not an international questionnaire, while it was developed to fit the Chinese cultural background. Also, this study didn't consider the interaction of social capital and other socio-economic factors.

## Conclusions

This research found a significant positive relationship between self-rated health and social capital. In the case of controlling factors such as health status and demographic characteristics, participants with higher social capital had better self-rated health. At the same time, we observed that urbanized rural residents with higher community trust and belonging had better self-rated health, however urban residents with higher personal social networks and family relationships had better self-rated health. The influence of social trust and sense of belonging on the health of urbanized rural residents cannot be ignored. In the process of urbanization, improving residents' social trust and sense of belonging will help urbanized rural residents better adapt to the new living environment and help them complete the identity transformation. In the future research on social capital, different types of communities should focus on the types of social capital from different sources, so as to take more targeted measures to improve the social support of residents and improve their health.

## Data Availability Statement

The raw data supporting the conclusions of this article will be made available by the authors, without undue reservation.

## Ethics Statement

The studies involving human participants were reviewed and approved by Institutional Review Board of School of Public Health, Sichuan University. The patients/participants provided their written informed consent to participate in this study.

## Author Contributions

TM and BG conceptualized the idea. TM collected the data, performed the statistical analyses, and wrote the first draft of the manuscript. BG critically revised the manuscript. All the authors checked and approved the final manuscript.

## Conflict of Interest

The authors declare that the research was conducted in the absence of any commercial or financial relationships that could be construed as a potential conflict of interest.

## Publisher's Note

All claims expressed in this article are solely those of the authors and do not necessarily represent those of their affiliated organizations, or those of the publisher, the editors and the reviewers. Any product that may be evaluated in this article, or claim that may be made by its manufacturer, is not guaranteed or endorsed by the publisher.
