# Supplementary material for: The Association of Social Capital and Self-Rated Health Between Urban Residents and Urbanized Rural Residents in Southwest China
Source: Front Public Health. 2021 Aug 25;9:718793. doi: 10.3389/fpubh.2021.718793 (PMC8425509; doi:10.3389/fpubh.2021.718793)
Supplement: Supplementary file 1 [file Table_1.DOCX]

Supplementary Material

# Table S1 Questionnaire

| **Part 1 Demographic characteristics** | |
| --- | --- |
| 1. Gender: 1) Male 2) Female |  |
| 2. Age: |  |
| 3. Marital status:  1) Single 2) Married 3) Divorced 4) Widowed |  |
| 4. Education status:  1) Primary school and below 2) Junior school 3) High school 4) College and above |  |
| 5. What is your average monthly income?  1) <2000 yuan 2) 2000～2999 yuan 3) 3000～3999 yuan 4) 4000+ yuan |  |
| 6. Employment status  1) Employed 2) Retired 3) Unemployed |  |
| **Part 2 Lifestyle factors and health status** | |
| 1. Are you currently smoking? 1) No 2) Yes |  |
| 2. Are you currently drinking alcohol? 1) No 2) Yes |  |
| 3. How many times have you exercised on average every week for the past six months?  1) 6 times and above 2) 1~5 times 3) <1 time |  |
| 4. Whether your body is sick or not within the two weeks before the investigation?  1) No 2) Yes |  |
| 5. Do you have a chronic disease diagnosed by a doctor?  1) No 2) Yes |  |
| 6. In general, what do you think of your health?  1) Excellent 2) Very good 3) Good 4) Fair 5) Poor |  |
| **Part 3 Social capital** | |
| **SC1: Personal social network** |  |
| 1. The number of people who are close to each other in daily life and economically. |  |
| 2. Locally, the number of people who are close to each other in daily life and economically. |  |
| 3. Number of social activities with non-family members in the last month.  1) At least 3 times a week 2) 1-2 times a week 3) less than 1 time a week 4) never |  |
| 4. Among the people who are close to you, how many people do you trust the most?  1) Trust yourself only  2) Trust one person only  3) At least two of the most trustworthy people  4) At least three of the most trustworthy people |  |
| **SC2: Interpersonal support** |  |
| 1. When you are sick or uncomfortable, can you always get the care of others?  1) Usually 2) Often 3) Sometimes 4) Occasionally 5) Never |  |
| 2. Do you have mutual help with residents in your daily life?  1) Usually 2) Often 3) Sometimes 4) Occasionally 5) Never |  |
| 3. Can the family provide emotional support for you?  1) Usually 2) Often 3) Sometimes 4) Occasionally 5) Never |  |
| 4. Can the family provide financial support for you?  1) Usually 2) Often 3) Sometimes 4) Occasionally 5) Never  **SC3: Family relationships** |  |
| 1. Who do you live with? 1) Not living alone 2) Living alone |  |
| 2. Is your marital status family structure intact? 1) No 2) Yes |  |
| 3. How is your relationship with your spouse?  1) Very good 2) Good 3) Fair 4) Not very good 5) Very bad 6) Not applicable |  |
| 4. How is your relationship with your parents?  1) Very good 2) Good 3) Fair 4) Not very good 5) Very bad 6) Not applicable |  |
| 5. How is your relationship with your children?  1) Very good 2) Good 3) Fair 4) Not very good 5) Very bad 6) Not applicable |  |
| **SC4: Community participation** |  |
| 1. Are you a member of a social activity organization? |  |
| 2. How often did you participate in a social activity in the last year?  1) Never 2) Occasionally 3) Sometimes 4) Often 5) Usually |  |
| 3. Have you received any help from the community in the past year? 1) No 2) Yes |  |
| **SC5: Community trust and belonging** |  |
| 1. Do you think that when you enter the community you will feel at home?  1) Very agree 2) Agree 3) General 4) Disagree 5) Very disagree |  |
| 2. If you have to move away from where you live now, would you feel sorry for it?  1) Very agree 2) Agree 3) General 4) Disagree 5) Very disagree |  |
| 3. Are you very interested in what happened in the residential area?  1) Very agree 2) Agree 3) General 4) Disagree 5) Very disagree |  |
| 4. If the community organizes volunteer activities, are you willing to participate?  1) Very willing 2) Willing 3) Don’t matter 4) Not verywilling 5) Unwilling |  |
| 5. Do you agree that “in general, people living in the community are trustworthy”?  1) Most people, whether they know or don’t know, are trustworthy.  2) Only the talents you know are trustworthy.  3) Only some people you know are trustworthy.  4) Everyone is not trustworthy |  |
| 6. To what extent do you trust the neighborhood committee?  1) Very trust 2) Trust 3) General 4) Not very trust 5) Don’t trust |  |
| 7. Do you agree that the security of your community is good?  1) Very agree 2) Agree 3) General 4) Disagree 5) Very disagree |  |

# Table S2 Relationship between SES characteristics and lower social capital

|  | SC1, n (%) | SC2, n (%) | SC3, n (%) | SC4, n (%) | SC5, n (%) |
| --- | --- | --- | --- | --- | --- |
| Residents type |  |  |  |  |  |
| Urban | 442(49.9) | 401(45.3) | 365(41.2) | 410(46.3) | 477(53.9) |
| Urbanized | 362(47.6) | 321(42.2) | 358(47.0) | 382(50.2) | 337(44.3) |
| *P* | 0.348 | 0.213 | 0.019* | 0.125 | <0.001** |
| Gender |  |  |  |  |  |
| Male | 263(51.5) | 222(43.4) | 204(39.9) | 220(43.1) | 291(56..9) |
| Female | 541(47.7) | 500(44.1) | 519(45.7) | 572(50.4) | 523(46.1) |
| *P* | 0.166 | 0.830 | 0.032* | 0.007* | <0.001** |
| Age(years) |  |  |  |  |  |
| <45 | 123(57.5) | 103(48.1) | 90(42.1) | 96(44.9) | 160(74.8) |
| 45~ | 208(49.3) | 182(43.1) | 106(25.1) | 225(53.3) | 228(54.0) |
| 55~ | 169(43.4) | 168(43.2) | 176(45.2) | 181(46.5) | 171(44.0) |
| 65~ | 304(49.0) | 269(43.3) | 351(56.5) | 290(46.7) | 255(41.1) |
| *P* | 0.012* | 0.610 | <0.001** | <0.095** | <0.001** |
| Marital status |  |  |  |  |  |
| Unmarried | 178(49.7) | 173(48.3) | 347(96.9) | 177(49.4) | 194(54.2) |
| Married | 626(48.6) | 549(42.6) | 376(29.2) | 615(47.7) | 620(48.1) |
| *P* | 0.720 | 0.062 | <0.001** | 0.591 | 0.049* |
| Education |  |  |  |  |  |
| Primary school and below | 332(52.3) | 285(44.9) | 369(58.1) | 373(58.7) | 288(45.4) |
| Junior school | 219(46.3) | 212(44.8) | 173(36.6) | 221(46.7) | 219(46.3) |
| High school | 137(46.1) | 130(43.8) | 101(34.0) | 120(40.4) | 153(51.5) |
| College and above | 116(48.1) | 95(39.4) | 80(33.2) | 78(32.4) | 154(63.9) |
| *P* | 0.161 | 0.498 | <0.001** | <0.001** | <0.001** |
| Monthly income(RMB) |  |  |  |  |  |
| <2000~ | 443(51.9) | 354(41.5) | 420(49.0) | 449(52.6) | 403(47.2) |
| 2000~ | 118(43.2) | 132(48.4) | 127(46.5) | 137(50.2) | 110(40.3) |
| 3000~ | 92(43.4) | 99(46.7) | 92(43.4) | 94(44.3) | 103(48.6) |
| 4000+ | 151(49.2) | 137(44.6) | 84(27.4) | 112(36.5) | 198(64.5) |
| *P* | 0.028* | 0.171 | <0.001** | <0.001** | <0.001** |
| Employment status |  |  |  |  |  |
| Employed | 243(51.7) | 222(47.2) | 170(36.2) | 230(48.9) | 302(64.3) |
| Retired | 194(41.8) | 214(46.1) | 210(45.3) | 174(37.5) | 190(40.9) |
| Unemployed | 367(51.5) | 286(40.2) | 343(48.2) | 388(54.5) | 322(45.2) |
| *P* | 0.002* | 0.029* | <0.001** | <0.001** | <0.001** |

Notes: *P<0.05, **P <0.001. SC1: Personal social network; SC2: Interpersonal support; SC3: Family relationship; SC4: Community participation; SC5: Community trust and belonging.

# Table S3 The distribution of health status, self-rated health and social capital among urban residents by gender

|  | Male (%) | Female (%) | $\chi^{2}$ | P |
| --- | --- | --- | --- | --- |
| Current smoker |  |  | 387.354 | <0.001** |
| No | 134(42.5) | 564(98.9) |  |  |
| Yes | 181(57.5) | 6(1.1) |  |  |
| Current drinker |  |  | 148.796 | <0.001** |
| No | 179(56.8) | 522(91.6) |  |  |
| Yes | 136(43.2) | 48(8.4) |  |  |
| Exercise/week(times) |  |  | 4.978 | 0.083 |
| 6+ | 181(57.5) | 369(64.7) |  |  |
| 1~5 | 64(20.3) | 89(15.6) |  |  |
| <1 | 70(22.2) | 112(19.6) |  |  |
| Chronic disease |  |  | 0.553 | 0.299 |
| No | 213(67.6) | 374(65.6) |  |  |
| Yes | 102(32.4) | 196(34.4) |  |  |
| Ill within the past 2 weeks | |  | 1.120 | 0.327 |
| No | 222(70.5) | 382(67.0) |  |  |
| Yes | 93(29.5) | 188(33.0) |  |  |
| Self-rated health |  |  | 6.238 | 0.014* |
| Good | 239(75.9) | 387(67.9) |  |  |
| Bad | 76(24.1) | 183(32.1) |  |  |
| SC1 |  |  | 1.162 | 0.293 |
| High | 150(47.6) | 293(51.4) |  |  |
| Low | 165(52.4) | 277(48.6) |  |  |
| SC2 |  |  | 3.749 | 0.057 |
| High | 186(59.0) | 298(52.3) |  |  |
| Low | 129(41.0) | 272(47.7) |  |  |
| SC3 |  |  | 3.938 | 0.054 |
| High | 199(63.2) | 321(56.3) |  |  |
| Low | 116(36.8) | 249(43.7) |  |  |
| SC4 |  |  | 3.315 | 0.078 |
| High | 182(57.8) | 293(51.4) |  |  |
| Low | 133(42.2) | 277(48.6) |  |  |
| SC5 |  |  | 12.617 | <0.001** |
| High | 120(38.1) | 288(50.5) |  |  |
| Low | 195(61.9) | 282(49.5) |  |  |

Notes: *P<0.05, **P <0.001. SC1: Personal Social Network, SC2: Interpersonal Support, SC3: Family Relationship, SC4: Community Participation, SC5: Community Trust and Belonging. Use the median as a criterion for dividing high and low group of social capital.

# Table S4 The distribution of health status, self-rated health and social capital among urbanized rural residents by gender

|  | Male (%) | Female (%) | $\chi^{2}$ | *P* |
| --- | --- | --- | --- | --- |
| Current smoker |  |  | 358.912 | <0.001** |
| No | 65(33.2) | 543(96.1) |  |  |
| Yes | 131(66.8) | 22(3.9) |  |  |
| Current drinker |  |  | 108.872 | <0.001** |
| No | 110(56.1) | 508(89.9) |  |  |
| Yes | 86(43.9) | 57(10.1) |  |  |
| Exercise/week(times) |  |  | 4.202 | 0.122 |
| 6+ | 122(62.2) | 396(70.1) |  |  |
| 1~5 | 31(15.8) | 74(13.1) |  |  |
| <1 | 43(21.9) | 95(16.8) |  |  |
| Chronic disease |  |  | 1.213 | 0.307 |
| No | 127(64.8) | 341(60.4) |  |  |
| Yes | 69(35.2) | 224(39.6) |  |  |
| Ill within the past 2 weeks | |  | 7.928 | 0.006** |
| No | 141(71.9) | 343(60.7) |  |  |
| Yes | 55(28.1) | 222(39.3) |  |  |
| Self-rated health |  |  | 2.592 | 0.118 |
| Good | 137(69.9) | 359(63.5) |  |  |
| Bad | 59(30.1) | 206(36.5) |  |  |
| SC1 |  |  | 0.626 | 0.455 |
| High | 98(50.0) | 301(53.3) |  |  |
| Low | 98(50.0) | 264(46.7) |  |  |
| SC2 |  |  | 3.004 | 0.093 |
| High | 103(52.6) | 337(59.6) |  |  |
| Low | 93(47.4) | 228(40.4) |  |  |
| SC3 |  |  | 0.488 | 0.507 |
| High | 108(55.1) | 295(52.2) |  |  |
| Low | 88(44.9) | 270(47.8) |  |  |
| SC4 |  |  | 3.564 | 0.068 |
| High | 109(55.6) | 270(47.8) |  |  |
| Low | 87(44.4) | 295(52.2) |  |  |
| SC5 |  |  | 2.359 | 0.133 |
| High | 100(51.0) | 324(57.3) |  |  |
| Low | 96(49.0) | 241(42.7) |  |  |

Notes: **P*<0.05, ***P* <0.001. SC1: Personal Social Network, SC2: Interpersonal Support, SC3: Family Relationship, SC4: Community Participation, SC5: Community Trust and Belonging. Use the median as a criterion for dividing high and low group of social capital.

# Table S5 Hosmer-Lemeshow test for the goodness-of-fit of the models

| Self-rated health | Model 1 | |  | Model 2 | |  | Model 3 | |
| --- | --- | --- | --- | --- | --- | --- | --- | --- |
|  | $\chi^{2}$ | *P* |  | $\chi^{2}$ | *P* |  | $\chi^{2}$ | *P* |
| Urban residents | 1.944 | 0.983 |  | 8.075 | 0.426 |  | 3.800 | 0.875 |
| Urbanized rural residents | 4.174 | 0.841 |  | 5.141 | 0.742 |  | 11.611 | 0.169 |

Notes: *P*>0.05 represents a good fit of the models.
